# LINC01116 facilitates colorectal cancer cell proliferation and angiogenesis through targeting EZH2-regulated TPM1

**DOI:** 10.1186/s12967-021-02707-7

**Published:** 2021-01-26

**Authors:** Weijie Liang, Jie Wu, Xinguang Qiu

**Affiliations:** 1 Department of Gastrointestinal Surgery, The First Affiliated Hospital of Zhengzhou University, Zhengzhou, Henan 450000 P.R. China; 2 Department of Ultrasound Intervention Surgery, The First Affiliated Hospital of Zhengzhou University, Zhengzhou, Henan 450000 P.R. China; 3 Department of Thyroid Surgery, The First Affiliated Hospital of Zhengzhou University, No. 1, East Jianshe Road, Erqi District, Zhengzhou, Henan 450000 P.R. China

**Keywords:** LINC01116, TPM1, Colorectal cancer, EZH2, Methylation, Proliferation, Angiogenesis

## Abstract

**Background:**

Colorectal cancer (CRC) is a common malignant tumor globally. Meanwhile, LINC01116 has been proposed as risk factor for various tumors, including CRC. But the regulation of LINC01116 in CRC required more validated data. This study aimed to elucidate the potential function of LINC01116 in regulating cell proliferation and angiogenesis of CRC.

**Methods:**

LINC01116 expression in 80 pairs of CRC tumor and adjacent non-tumor tissues was determined by qRT-PCR. After transfection of pcDNA3.1-LINC01116, sh-LINC01116, sh-TPM1, pcDNA3.1-EZH2 or sh-EZH2 in SW480 and HCT116 cells, the levels of LINC01116, TPM1 and EZH2 were measured by qRT-PCR or Western blot. The cell biological function of CRC cell lines was determined by CCK-8, colony formation assays, tube formation and scratch assays. RNA pull-down and RIP assays were applied to detect the binding of LINC01116 with EZH2 and H3K27me3. Binding of EZH2 to the TPM1 promoter was assessed by ChIP assay. Finally, xenograft models in nude mice were established to validate the results of in vitro experiments.

**Results:**

LINC01116 was overexpressed in CRC tissues and high expression of LINC01116 was negatively correlated with postoperative survival. In vitro study showed LINC01116 expression could significantly enhance CRC progression, including increasing cell proliferation, migration and angiogenesis. Besides, investigations into the mechanism disclosed that LINC01116 could regulate EZH2 to inactivate TPM1 promoter, thus promoting CRC cell proliferation and angiogenesis. Moreover, consistent results of in vivo experiments were conformed in vitro experiments.

**Conclusion:**

LINC01116 promotes the proliferation and angiogenesis of CRC cells by recruiting EZH2 to potentiate methylation in the TPM1 promoter region to inhibit the transcription of TPM1.

## Background

As one of the most common malignancies worldwide, colorectal cancer (CRC) accounts for nearly 8.5% of the total cancer-related deaths [1]. Although surgical resection is currently the optimal treatment approach for CRC, the 5-year survival rate for patients with late-stage CRC is less than 10% [2, 3]. Tumor angiogenesis is crucial for sustained cell metastasis and tumor proliferation [4]. To date, suppression of tumor angiogenesis is one of the important strategies to hinder tumor metastasis and growth. However, traditional anti-angiogensic therapies sometimes induced hypoxia and metastasis which might speed up the growth of tumor cells [5, 6]. To maximize the efficiency of anti-angiogensic therapies in CRC, identification of modulatory mechanisms that regulate angiogenesis is essential for CRC treatment. Although increasing studies have been emerged to elucidate the underlying molecular mechanisms in tumorigenesis and CRC progression, the identification of potential new therapeutic targets for intervention of CRC is still necessary considering the absence of optimal therapeutic strategy.

Long non-coding RNAs (lncRNAs) are a recently discovered category of non-coding RNAs that longer than 200 nt without a protein-coding capacity [7, 8]. A growing number of researches have clarified that lncRNAs involve in a variety of cellular biological functions, including chromatin imprinting, cell differentiation, tumorigenesis, and cancer cell drug resistance [9, 10]. Importantly, lncRNAs as promising candidates for the diagnosis of CRC have been discovered [11–14]. Despite great efforts in understanding the occurrence and progression of CRC, the clinical trials of CRC treatments had disappointing results. Previously, it has been revealed that lncRNA LINC01116 is abnormally up-regulated in several types of cancer [15–17]. For instance, LINC01116 was expressed frequently in prostate cancer but was repressed after sulforaphane treatment [18]. In osteosarcoma, LINC01116 was reported as a potential disease risk factor closely associated with cancer development [17]. Besides, existing study reported that overexpression of LINC01116 promotes cell progression including angiogenesis of glioma [19] and in CRC [20], but more data are required for exploring the mechanism of LINC01116 regulation in CRC. Since the knowledge about lncRNAs is still limited, discovering novel CRC-related lncRNAs are extremely essential in order to better explore the etiology and pathology of this disease. In the present work, we mainly focus on the effects and mechanism of LINC01116 in the proliferation and tumorigenesis of CRC.

In the present report, we aimed to elucidate the clinical significance and potential molecular mechanism of LINC01116 in CRC. We disclosed that LINC01116 is overexpressed in human CRC tissues and that LINC01116 overexpression is strongly correlated with a poor prognosis. Additionally, LINC01116 overexpression in CRC cells stimulated proliferation and angiogenesis both in vivo and in vitro. Meanwhile, methylation was proved to have certain role to play in the development of CRC [21–23]. Therefore, we hypothesize the implication of LINC01116 in CRC may also have certain relationship with methylation. LINC01116 was found to bind to EZH2. Among all the downstream target of EZH2, tropomyosin 1 (TPM1) was proved to be repressed in CRC tissues evidenced by TCGA database [24]. Meanwhile, the mechanism of TPM1 in regulating CRC progression remains large to be determined. Mechanistically, the result in this study showed LINC01116 enhances proliferation and angiogenesis in CRC tumors through recruiting of EZH2 to downregulate TPM1 expression. This research suggest LINC01116 knockdown as a potential therapeutic strategy for CRC treatment and the mechanism herein involves the EZH2/TPM1 axis. LINC01116 may serve as a predictive biomarker and a potential therapeutic target in CRC treatment.

## Materials and methods

### Cell culture

The human CRC cell lines (SW480, SW1116, HT29, HCT116 and LoVo), normal colon epithelial cells (NCM 460) and human umbilical vein endothelial cells (HUVECs) were acquired from American Type Culture Collection (ATCC, Manassas, Virginia, USA). Cell lines were cultured in DMEM (Gibco, Grand Island, NY, USA) with 1% penicillin–streptomycin and 10% fetal bovine serum (FBS) at 37℃ with 5% CO_2_.

### Collection of clinical samples

A total of 80 cases of fresh CRC tissues and adjacent normal tissues were acquired from the First Affiliated Hospital of Zhengzhou University. Tissue samples were immediately cooled in liquid nitrogen. This study was ethically licensed by the local hospital ethics committee and written informed consent was obtained from all subjects.

### qRT-PCR

Total RNAs were extracted by using TRIzol reagent (Invitrogen, Carlsbad, CA, USA), and a reverse transcription kit (TaKaRa, Tokyo, Japan) was used for the reverse transcription. All operations were carried out according to the manufacturer’s instructions. Besides, gene expression was measured using the LightCycler 480 fluorescence quantitative PCR instrument (Roche, Indianapolis, IN, USA) and reaction condition was conducted utilizing the fluorescent quantitative RT-PCR kit (SYBR Green Mix, Roche Diagnostics, Indianapolis, IN). The thermocycling program were as follows: 10 s pre-denaturation at 95 ℃, followed by 45 cycles of 5 s denaturation at 95 ℃, 10 s annealing at 60 ℃, 10 s extension at 72 ℃, and a final extension at 72 ℃ for 5 min. Three replicates were set for each reaction of qPCR and GAPDH was used as internal reference. Data were analyzed using 2^−ΔΔCt^ method according to the following formula: ΔΔCt = [Ct _(target gene)—_Ct _(reference gene)_] _experimental group—_[Ct _(target gene)—_Ct _(reference gene)_] _control group_. The primer sequences for all candidate reference genes are listed in Table 1.

**Table 1:** Primer sequence for quantitative reverse transcription polymerase chain reaction to determine the expression levels of LINC01116, TPM1, EZH2 and GAPDH

Name of primer	Sequences
LINC01116-F	CCCGAGTACCTGACTGAGGA
LINC01116-R	TCCAAGCAGGGAGGTCAAAC
TPM1-F	ACAGAGGTGACTGAAACTGACA
TPM1-R	ATGTGGATGCCCTTGAATTGGA
EZH2-F	GGAGTAGCTTCGCCTCTGAC
EZH2-R	GGCAACTCACTAGGCGTTCA
GAPDH-F	GTGGCTGGCTCAGAAAAAGG
GAPDH-R	GGGGAGATTCAGTGTGGTGG

*F* forward, *R* reverse, *TPM1* tropomyosin 1

### Western blot

Cells were lysed by RIPA buffer to obtain the proteins. After, the protein concentration was measured by bicinchoninic acid assay (BCA) kit, the corresponding volume of protein was added into with loading buffer and then heated in boiling water (3 min). RIPA buffer, BCA kit, and loading buffer were obtained from Beyotime Institute of Biotechnology. After the protein was denatured, the electrophoresis was firstly performed for 30 min at 80 V, and then for 1–2 h at 120 V after bromophenol blue was into the separation gel. The membrane transfer was conducted on an ice bath for 60 min at 300 mA, after which the membrane was rinsed (1–2 min) then blocked for 60 min at room temperature, or sealed at 4 ℃ overnight. Primary antibody against EZH2 (5246S, 1:1000), TPM1 (3910S, 1:1000), GAPDH (5174S, 1:1000) or H3K27me3 (9733S, 1:1000) (all from Cell Signaling, Boston, USA) was cultured on a shaker for 1 h at room temperature, followed by washing for 3 × 10 min. Then, the membrane was incubated with secondary antibodies for 1 h at room temperature before 3 × 10 min washing. Finally, the membrane was visualized utilizing chemiluminescence imaging (Bio-Rad, Hercules, CA, USA).

### Cell transfection

pcDNA3.1-LINC01116, sh-LINC01116, sh-TPM1, pcDNA3.1-EZH2, sh-EZH2 and their negative controls were acquired from GenePharma (Shanghai, China). Lipofectamine 2000 transfection reagent (Invitrogen, Carlsbad, CA, USA) was used for transfection following the manufacturer’s protocol.

### CCK-8 assay

Twenty four hours after cell transfection, SW480 and HCT116 cells were inoculated into the 96-well plates, and cell suspensions (100 μl with 1 × 10^5^ cells/ml) were seeded into each well with three replicates per group. Thereafter, 10 μl of CCK-8 solution (Tokyo, Dojindo, Japan) was added into each well after incubation for 0, 24, 48, 72 and 96 h. Finally, the absorbance was determined at 450 nm after cells were cultured for another 2 h.

### Colony formation assay

After transfection for 24 h, the collected SW480 and HCT116 cells of each group were trypsinized and centrifuged (1500 rpm, 5 min, 25 ℃), after which the complete medium was added to resuspend cells. Afterwards, cells (500 cells/well) were inoculated into 6-well plates containing 2 ml of complete medium in an atmosphere of 5% CO_2_ at 37 ℃ for 2 to 3 weeks. The culture was terminated when clones in 6-well plates were visible to the naked eye. After culture medium was removed, cells were washed in PBS twice and then fixed with 1.5 ml of formaldehyde for 15 min. Thereafter, cells were stained with 1 ml of Giemsa solution for 20 min in the dark. Giemsa solution was washed away slowly by running water, and the plates were air-dried. Finally, the colonies were counted.

### Cell scratch test

Firstly, HUVECs (1 × 10^6^) were seeded into transwell upper chamber with pore size of 0.4 μm (Millipore, Billerica, MA, USA) without matrigel. Equal amounts of SW480 and HCT116 cells of each group following transfection were inoculated into the 24-well plates in the lower chamber. After 24 h co-culture (5% CO_2_, 37 ℃), HUVECs were used for the subsequent assays.

HUVEC cells were inoculated into 6-well plates with a density of 2 × 10^6^ /well and maintained in incubator (5% CO_2_, 37 ℃) for 24 h. Subsequently, cells grown in monolayer were scratched with a sterile 200 μl pipette tip, followed by PBS washing and subsequent incubation in 5% CO_2_ at 37 ℃ for 24 h. The scratch distance after culture for 0 h and 24 h was observed and photographed under a microscope. HUVEC cell migration rate was calculated as follows: Migration rate = (0 h scratch distance—24 h scratch distance)/0 h scratch distance.

### Tube formation assay

Before the experiment, matrigel (Corning, Tewksbury, MA, USA), 48-well plates (Millipore, Billerica, MA, USA), and the tips were pre-cooled or dissolved at 4 ℃. Next, a pre-cooled pipette tip was performed to transfer matrigel (100 μl) to the 48-well plate and cultured in an incubator for 30 min at 37℃. Furthermore, 2 × 10^4^ HUVECs were co-cultured with SW480 and HCT116 cells and then added into each well for cell culture of 6 h. Finally, images were captured by measuring the total vascular length of HUVECs with Image J (NIH, Bethesda, MD, USA).

### RNA immunoprecipitation (RIP) assay

RIP assay was performed to test the binding of LINC01116 to EZH2. The cells were collected and washed twice with pre-cooled PBS, followed by centrifugation (5 min, 1,500 rpm) and reaction with equal volume of RIP lysis buffer. Then, RIP Wash Buffer (100 μl) was used to re-suspend the magnetic beads, and 5 μg of EZH2 antibody (5246S, 1:100, Cell Signaling, Boston, USA) or IgG antibody was added to the tube for 30 min at room temperature. Thereafter, the centrifuge tube was placed on a magnetic rack with supernatant discarded and 500 μl of RIP Wash Buffer was added by vortexing for twice. After that, 500 μl of RIP Wash Buffer was added for vortex oscillation and placed on ice. The supernatant in magnetic bead tube was discarded after which 900 μl of RIP Immunoprecipitation Buffer was transferred into each tube. Then, cell lysates were centrifuged at 14,000 rpm for 10 min at 4 ℃. Afterwards, the magnetic bead-antibody complex was added with supernatant (100 μl) and incubated at 4 ℃ overnight for transient centrifugation with supernatant removed. Then, RIP Wash Buffer (500 μl) was used to wash the complex for vibration with the supernatant abandoned, followed by six washes. Each sample was re-suspended with 150 μl of Proteinase K Buffer magnetic bead-antibody complex and cultured for 30 min at 55 ℃. qRT-PCR was used to estimate the expression of LINC01116 after RNA was extracted.

### Chromatin immunoprecipitation (ChIP)

The binding of EZH2 to TPM1 promoter was analyzed by ChIP assays utilizing a ChIP Kit (Millipore, Billerica, MA, USA) according to manufacturer’s directions. Briefly, formaldehyde was used to investigate the crosslink between DNA and proteins and fixed for 30 min. Afterwards, the DNAs isolated from CRC cells were fragmented into 200 –1000 bp with sonication. Then, the subsided DNA fragments were determined by qRT-PCR following the fragmented DNAs were incubated with EZH2 or IgG antibody.

### RNA pull-down assay

RNA pull-down was used to measure proteins that bind to LINC01116. Briefly, biotin-dUTP-labeled LINC01116 probe (LINC01116 probe) or negative control probe (scram probe) was incubated with the cell lysate. Then, the proteins in complex were isolated and measured by Western blot.

### Animals

Twelve specific pathogen-free (SPF) BALB/c nude mice that weighed 16 ± 2 g and aged 4 – 6 weeks were provided by Shanghai SLAC Laboratory Animal Co., Ltd. and cultured in a SPF sterile laminar flow chamber with humidity (55 ± 5%) and constant temperature (22 –26 °C). All mice intraperitoneal injected with 50 mg/kg ketamine and 60 mg/kg sodium pentobarbital were randomly assigned into sh-NC group and sh-LINC01116 group. After that, nude mice inoculated subcutaneously with SW480 cells (5 × 10^5^) of stable lowly expressed LINC01116 or negative control SW480 cells (5 × 10^5^) were named as sh-LINC01116 group or sh-NC group, respectively. Following two weeks, the mice were sacrificed, and tumor volume and weight were calculated. Tumor volume = 0.5 × length × width^2^. Finally, the tumor tissues were paraffin embedded and sectioned.

### H&E staining

The tumor tissues were harvested and fixed in 4% paraformaldehyde for 48 h to prepare paraffin Sect. (4 μm thickness). After dehydration and clearing, the embedding, slicing, staining, and mounting schedules were carried out. Histopathological changes were observed under an optical microscope.

### Immunohistochemistry

After fixation with 4% paraformaldehyde for 48 h, the isolated tumor tissues were prepared into paraffin sections of 4 μm thickness. Then, the paraffin sections were baked for 20 min, followed by conventional xylene dewaxing and distilled water washing. Following three times washing with PBS, 3% H_2_O_2_ was added on the sections for 10 min at room temperature. Thereafter, sections were subjected to antigen retrieval after washing with PBS thrice. Afterwards, normal goat serum was added at room temperature for 20 min, and then the sections were incubated with primary antibody against CD31 (ab134168, 1:250) or Ki-67 (ab16667, 1:200) (Abcam, Cambridge, MA, USA) for overnight at 4℃, followed by PBS washing for three times. Then, sections were cultured for 1 h with secondary antibody at room temperature and washed three times with PBS, followed by diaminobenzidine development for 1 –3 min. Finally, the nuclei were stained with hematoxylin for 3 min, and routine dehydration, permeabilization and mounting were performed.

### Statistical analysis

Graphpad Prism 7 software was used for statistical analysis. All values are expressed as mean ± standard deviation. Two sets of data were conducted by *T*-test, while the differences among groups were monitored by One-way analysis of variance (ANOVA). Dunnett's multiple comparisons test was instructed to multiple comparisons after ANOVA. The associations of LINC01116 expression with overall survival of CRC patients were assessed by Kaplan–Meier analysis and the relation of LINC01116 expression to pathological characteristics of CRC patients was measured by chi-square test. *P* < 0.05 was deemed as statistically significant.

## Results

### LINC01116 is highly expressed in CRC tissues

**Fig. 1:**
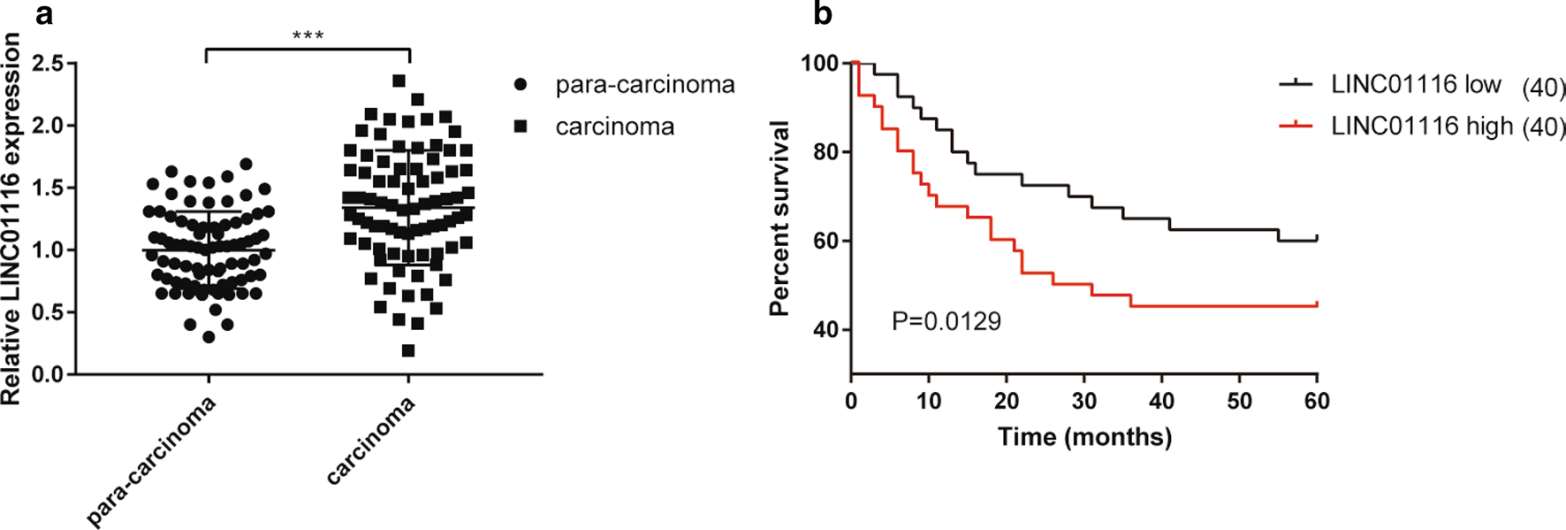
LINC01116 is increased in CRC tissues. qRT-PCR was used to determine the mRNA level of LINC01116 in CRC tissues and adjacent normal tissues, n = 80 (**a**). Effects of LINC01116 expression on prognosis of CRC patients were assessed by Kaplan–Meier analysis (**b**). ***P < 0.001 vs. para-carcinoma tissues; *CRC* colorectal cancer

To determine whether LINC01116 was dysregulated in CRC, qRT-PCR was applied to measure LINC01116 expression in 80 pairs of CRC tumor and adjacent normal tissues. As depicted in Fig. 1a, LINC01116 expression was upregulated in tumor tissues in comparison with adjacent normal tissues (*P* < 0.05). Moreover, we analyzed the association of clinicopathologic features of each included patients with the LINC01116 expression level. Patients with LINC01116 expression above median were classified as LINC01116 High group (n = 40) while that of below median were as LINC01116 Low group (n = 40). As shown in Table 2, Chi-square test showed the levels of LINC01116 in CRC tissues was significantly correlated with histological differentiation and tumor size (*P* < 0.05), and no significant relationship was found with patient’s age, gender, TNM staging and lymphatic metastasis. Kaplan–Meier analysis revealed that LINC01116-high patients had lower 5-year survival rates than those of LINC01116-low patients (Fig. 1b, P < 0.05). All these data hinted that the expression level of LINC01116 was upregulated in CRC tissues and was determined to have oncogenic activity.

**Table 2:** The association of LINC01116 expressions with the clinical pathological characteristics of CRC patients

Pathological characteristics	Low expression of LINC01116 (n = 40)	High expression of LINC01116 (n = 40)	*P* values
Gender (F/M)	18/22	20/20	0.8230
Age (< 60/ ≥ 60)	11/29	14/26	0.6300
Tumor size (< 3/ ≥ 3 cm)	33/7	23/17	0.0270*
TNM (I-II/III-IV)	18/22	21/19	0.6549
Differentiated degree (poor/moderate or well)	3/37	11/29	0.0367*
Lymph node metastasis (no / yes)	22/18	24/16	0.8213

*P < 0.05, f female, *m* male, *TNM* tumor lymph node metastasis, *CRC* colorectal Cancer

### LINC01116 overexpression stimulates CRC cell proliferation and angiogenesis

**Fig. 2:**
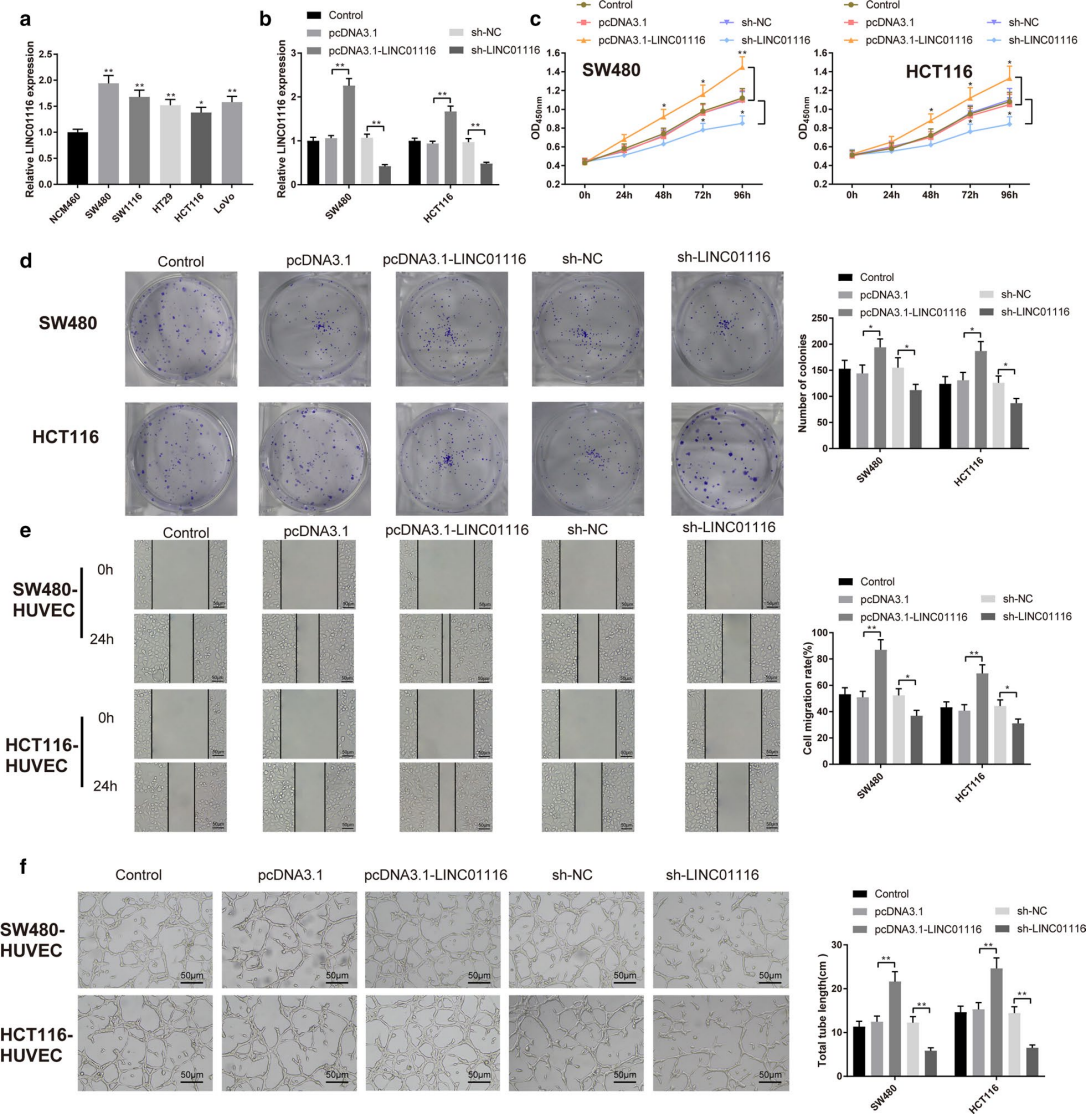
LINC01116 promotes CRC cell proliferation and angiogenesis**.** qRT-PCR was employed to measure LINC01116 expression in CRC cell lines (**a**). After pcDNA3.1-LINC01116 or sh-LINC01116 was transfected into SW480 and HCT116 cells, the mRNA of LINC01116 was measured by qRT-PCR (**b**). CCK-8 assay was used to determine cell viability (**c**), and colony formation assay to determine cell proliferation (**d**). Migration and angiogenesis were monitored by cell scratch (**e**) and tube formation assays (**f**), respectively. *P < 0.05, **P < 0.01; *CRC* colorectal cancer

This step was to ascertain the regulative role of LINC01116 in human CRC cell lines and normal colon epithelial cells. Analysis using qRT-PCR showed that LINC01116 was expressed abundantly in CRC cells as compared to NCM460 cells (Fig. 2a, P < 0.05). Next, pcDNA3.1-LINC01116 or sh-LINC01116 was transfected into SW480 and HCT116 cells to measure the levels of LINC01116. As depicted in Fig. 2b, LINC01116 was highly expressed in pcDNA3.1-LINC01116 group (vs. pcDNA3.1 group) and lowly expressed in sh-LINC01116 group (vs. sh-NC), while the mRNA level of LINC01116 in pcDNA3.1 and sh-NC groups was not significantly different from that in Blank group, indicating for satisfactory transfection efficiency of LINC01116 overexpression and knockdown in CRC cells.

Tube formation and scratch assays were conducted to examine migration and angiogenesis of HUVECs co-transfected with SW480 or HCT116 cells. The detection revealed that elevated cell proliferation activity and number of clones were found in pcDNA3.1-LINC01116 group (pcDNA3.1 group), and suppressed cell proliferation activity and number of clones in sh-LINC01116 group (vs. sh-NC group) (Fig. 2c, d, P < 0.05). Meanwhile, cell migration and total tube length of HUVECs in pcDNA3.1-LINC01116 group were strengthened obviously as compared to pcDNA3.1 group, while that of in sh-LINC01116 group behaved in the opposite fashion (vs. sh-NC group) (Fig. 2e, f, P < 0.05). Above data implies that LINC01116 expression was highly elevated on CRC cells and potentiated CRC cell proliferation and angiogenesis.

### LINC01116 regulating TPM1 to potentiate CRC cell proliferation and angiogenesis

**Fig. 3:**
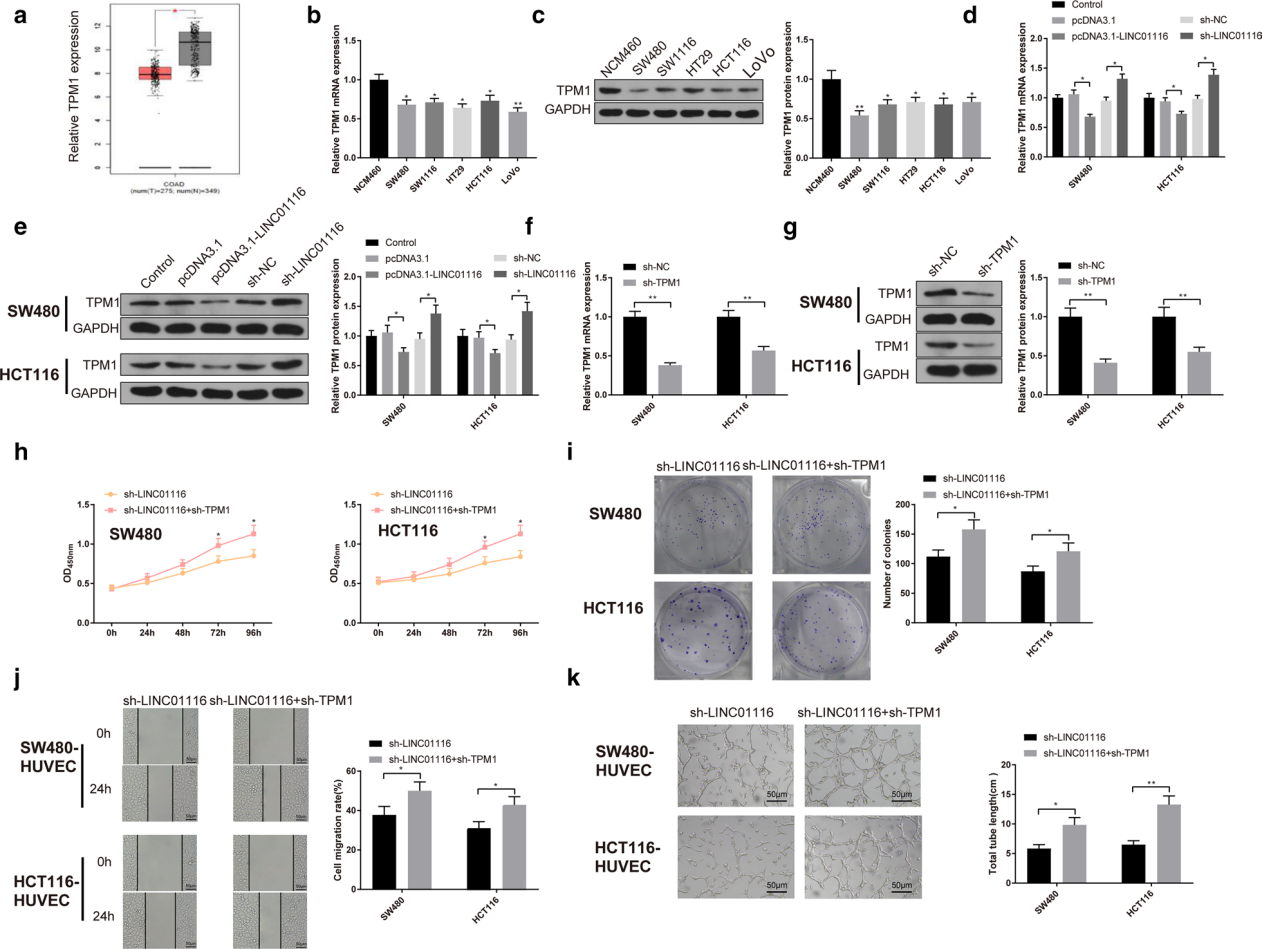
LINC01116 accelerates CRC cell proliferation and angiogenesis by mediating TPM1 Note: The expression of TPM1 in CRC tissues of TCGA dataset was searched through GEPIA database (**a**). The levels of TPM1 in CRC cells were tested by qRT-PCR (**b**) and Western blot (**c**). After transfection of pcDNA3.1-LINC01116 or sh-LINC01116 into SW480 and HCT116 cells, qRT-PCR (**d**) and Western blot (**e**) were applied to test the levels of TPM1. After SW480 and HCT116 cells were transfected with sh-TPM1, the levels of TPM1 were tested by qRT-PCR (**f**) and Western blot (**g**). Cell viability and proliferation were assessed by CCK-8 (**h**) and colony formation assays (**i**). Cell scratch (**j**) and tube formation assays (**k**) were used to measure migration and angiogenesis. *P < 0.05, **P < 0.01; *CRC* colorectal cancer, *TPM1* tropomyosin 1

Analysis of the GEPIA database (http://gepia.cancer-pku.cn/index.html) showed that TPM1 was poorly expressed in CRC tissues in TCGA dataset (Fig. 3a, P < 0.05). Moreover, qRT-PCR and Western blot also corroborated that TPM1 was lowly expressed in CRC cells (Fig. 3b, c, P < 0.05). Meanwhile, TPM1 was downregulated following overexpression of LINC01116 while upregulated after knockdown of LINC01116 in SW480 and HCT116 cells (Fig. 3d, e, P < 0.05). Herein, we speculated that LINC01116 promotes cell progression in CRC in TPM1-dependent manner.

As shown in Fig. 3f, g, TPM1 was downregulated strikingly following transfection of sh-TPM1 (*P* < 0.05), indicating for satisfactory transfection efficiency of TPM1 knockdown. Then, sh-LINC01116 or sh-LINC01116 + sh-TPM1 were transfected in SW480 and HCT116 cells to measure CRC cell proliferation and angiogenesis. The results showed that SW480 and HCT116 cell proliferation activity and number of clones of sh-LINC01116 + sh-TPM1 group were increased compared with sh-LINC01116 group (Fig. 3h, i, P < 0.05). sh-LINC01116 + sh-TPM1 group had elevated cell migration ability and total tube length than sh-LINC01116 group (vs. sh-LINC01116 group) (Fig. 3j,k, P < 0.05). Taken together, the data suggested that knockdown of TPM1 could abandon LINC01116 silencing induced tumor inhibition. All in all, LINC01116 facilitates CRC cell proliferation and tumor angiogenesis by negatively regulating TPM1 expression.

### LINC01116 recruits EZH2 to inhibit TPM1 gene transcription

**Fig. 4:**
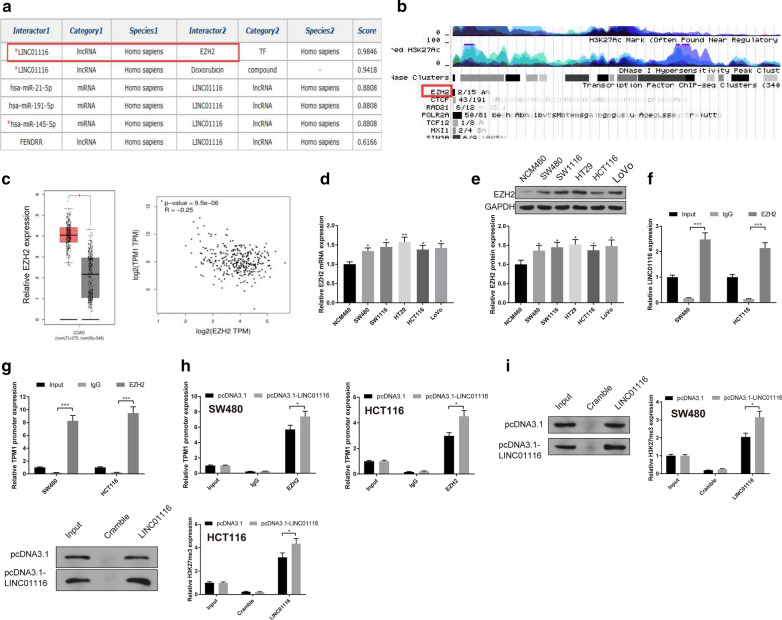
LINC01116 recruits EZH2 to suppress TPM1 gene transcription**.** The binding of LINC01116 and EZH2 were predicted by RNAInter online tools (**a**). The potential binding of EZH2 and TPM1 was predicted by UCSC (**b**). TCGA database was used to assess the expression of EZH2 in CRC tissues and the association between EZH2 and TPM1 (**c**). qRT-PCR (**d**) and Western blot (**e**) were applied to measure the levels of EZH2 in CRC cell lines. RIP assay confirmed the interaction between LINC01116 and EZH2 (**f**). Binding of EZH2 to TPM1 promoter was verified by ChIP (**g**). The augment of TPM1 promoter enriched in EZH2 antibody following overexpression of LINC01116 was detected by ChIP (**h**). RNA pull-down assay was performed to measure H3K27me3 in LINC01116-complex following overexpression of LINC01116 (**i**). *P < 0.05, **P < 0.01, ***P < 0.001; *TPM1* tropomyosin 1, *CRC* colorectal cancer

To ascertain the mechanism by which LINC01116 reversely regulates TPM1 expression, RNAInter (http://www.rna-society.org/raid/search.html) predicted protein that binds to LINC01116. As shown in Fig. 4a, LINC01116 contains binding site to EZH2. Besides, UCSC online tool (http://genome.ucsc.edu/cgi-bin/hgNear) confirmed that EZH2 is a transcription factor for TPM1 (Fig. 4b). Moreover, TCGA database revealed that EZH2 was expressed abundantly in CRC tissues and negatively regulates TPM1 (Fig. 4c, P < 0.05). qRT-PCR and Western blot were further tested that EZH2 was strongly expressed in SW480 and HCT116 cells (Fig. 4d, e, P < 0.05). The combination of LINC01116 and EZH2 was verified by RIP. The detection showed that the complex pulled down by EZH2 antibody had enriched LINC01116 expression (Fig. 4f, P < 0.05), suggesting LINC01116 could bind with EZH2. Meanwhile, ChIP assay disclosed that the complex pulled down by the EZH2 antibody also enriched the TPM1 promoter (Fig. 4g, P < 0.05), indicating EZH2 is a transcription factor of TPM1. After overexpression of LINC01116, the TPM1 promoter in complex pulled down by EZH2 antibody was increased significantly (Fig. 4h, P < 0.05). RNA pull-down results showed the protein of H3K27me3 was obviously increased (Fig. 4i, P < 0.05), and the level of TPM1 strikingly decreased in pcDNA3.1-LINC01116 group (Fig. 3d, e, P < 0.05). The aforementioned results corroborated that LINC01116 could recruit EZH2 to promote the methylation of TPM1, thus inhibiting the transcription of TPM1.

### EZH2 inhibits TPM1 to promote CRC cell proliferation and angiogenesis

**Fig. 5:**
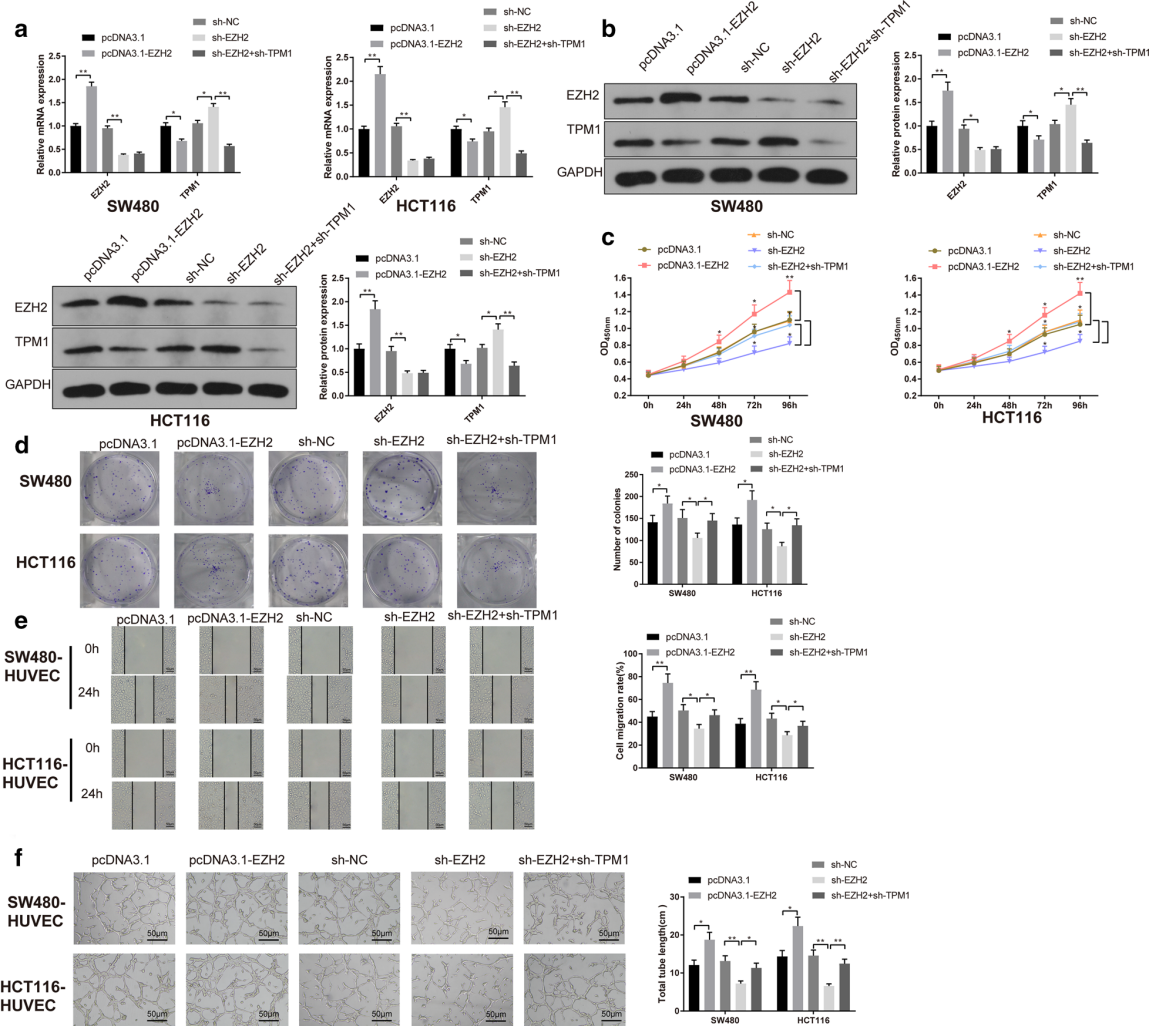
EZH2 inhibits TPM1 to promote CRC cell proliferation and angiogenesis**.** After SW480 and HCT116 cells were transfected with pcDNA3.1- EZH2, sh- EZH2 or sh-EZH2 + sh-TPM1, qRT-PCR (**a**) and Western blot (**b**) were employed to measure the levels of EZH2 and TPM1. Cell viability, proliferation, migration and angiogenesis were detected by CCK-8 assay (**c**), colony formation assay (**d**), cell scratch (**e**), tube formation assays (**f**), respectively. *P < 0.05, **P < 0.01; *TPM1* tropomyosin 1, *CRC* colorectal cancer

We further explored the effect of EZH2 on CRC cell proliferation and angiogenesis through inhibiting TPM1 expression. Firstly, pcDNA3.1-EZH2, sh-EZH2 or sh-EZH2 + sh-TPM1 was transfected/co-transfected into SW480 and HCT116 cells. Analyses by qRT-PCR and Western blot manifested that EZH2 was strongly expressed and TPM1 was lowly expressed in pcDNA3.1-EZH2 group (vs. pcDNA3.1 group). While sh-EZH2 group had refrained expression of EZH2 and heightened expression of TPM1 than in sh-NC group (Fig. 5a, b, P < 0.05). Above results supported a high transfection efficiency of EZH2 and indicated that EZH2 could negatively regulate TPM1 expression.

Further, CCK-8 and colony formation assays uncovered that SW480 and HCT116 cell proliferation and colony numbers were strengthened in pcDNA3.1-EZH2 group (vs. pcDNA3.1 group), repressed in sh-EZH2 group (vs. sh-NC group), and elevated in sh-EZH2 + sh-TPM1 group (vs. sh-EZH2 group) (Fig. 5c, d, P < 0.05).

Additionally, tube formation and scratch assays manifested that HUVEC cell migration and total tube length were heightened in pcDNA3.1-EZH2 group (vs. pcDNA3.1 group), weakened in sh-EZH2 group (vs. sh-NC group), and enhanced in sh-EZH2 + sh-TPM1 group (vs. sh-EZH2 group) (Fig. 5e, f, P < 0.05). The collective data indicated that EZH2 blocks TPM1 expression to promote CRC cell proliferation and angiogenesis.

### LINC01116 promotes tumor growth and angiogenesis in vivo

**Fig. 6:**
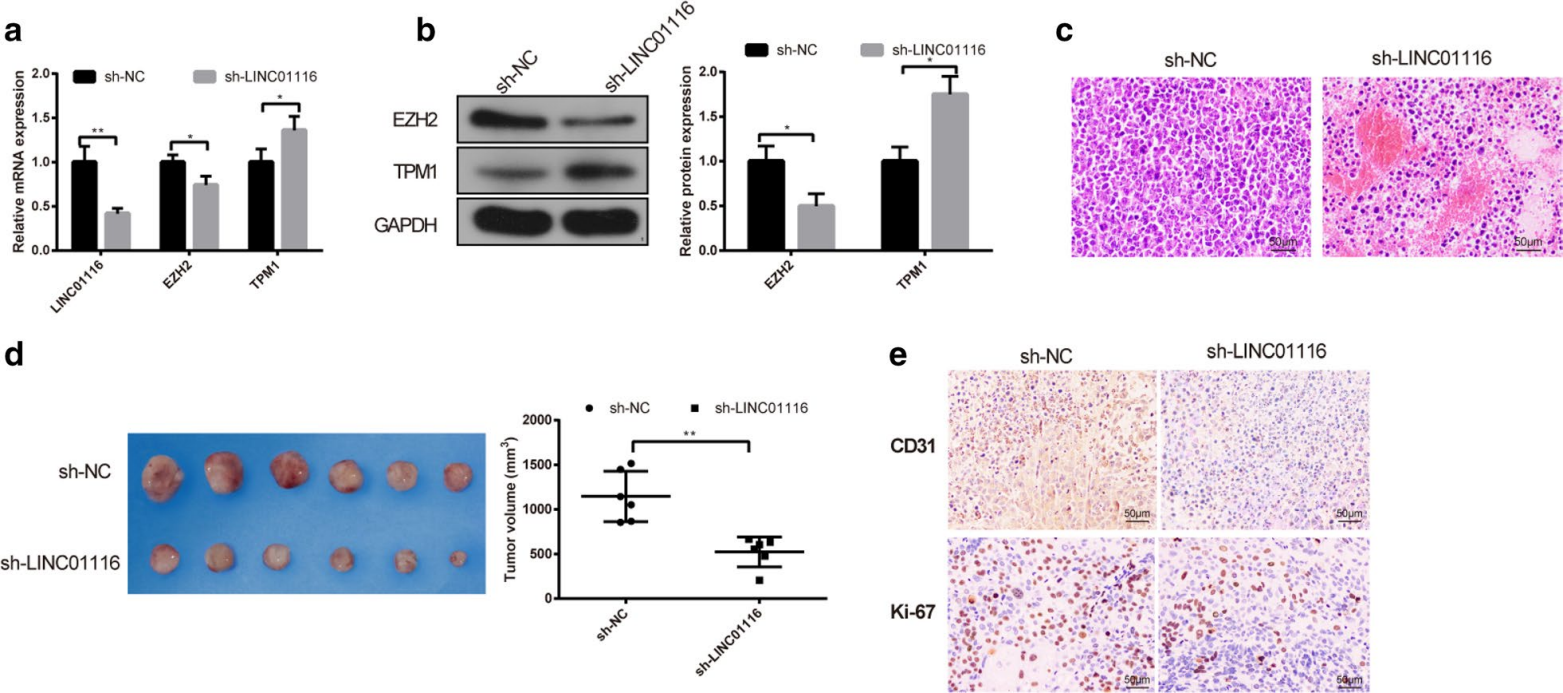
LINC01116 promotes tumor growth and angiogenesis in vivo. After nude mice were injected with SW480 cells that transfected with sh-LINC01116 or sh-NC, the levels of LINC01116, EZH2 and TPM1 in tumor tissues were tested by qRT-PCR (**a**) and Western blot (**b**), n = 6. Tumor histopathology was detected by H&E staining (**c**), n = 6. The tumor size in xenograft model was calculated (**d**), n = 6. IHC was used to detect CD31 and Ki-67 expressions in tumor tissues (**e**), n = 6. *P < 0.05, **P < 0.01; *TPM1* tropomyosin 1

The effect of LINC01116 on tumor growth and angiogenesis in nude mice were assessed. Briefly, SW480 cells were injected into nude mouse to construct xenograft models, and the tumor formation rate was 100%. RT-PCR and Western blot revealed that sh-LINC01116 group had decreased levels of LINC01116 and EZH2 and increased levels of TPM1 (vs. sh-NC group) (Fig. 6a, b, P < 0.05). H&E staining indicated that tumor cell density was obviously reduced in the sh-LINC01116 group (vs. sh-NC group), and sh-LINC01116 group showed a massive tumor-cell necrosis in tumor tissues (Fig. 6c). After two weeks, the tumor volume was effectively smaller in sh-LINC01116 group than in sh-NC group (Fig. 6d, P < 0.05). Further, expressions of CD31 and Ki-67 in tumor tissues were detected by IHC. The results disclosed that the expressions of CD31 and Ki-67 were repressed in sh-LINC01116 group when compared with sh-NC group (Fig. 6e, P < 0.05). Collectively, knockdown of LINC01116 impeded tumor growth and angiogenesis in vivo.

## Discussion

LINC01116 plays oncogenic roles in multiple cancer types [15, 17, 25], while only one previous study reported its involvement in CRC [20]. The mechanism of LINC01116 regulation in CRC is still far from understanding. Our findings described that LINC01116 is excessively expressed in CRC tumor tissues and is correlated with a poor prognosis. Mechanistically, LINC01116 accelerates CRC proliferation and angiogenesis via regulating EZH2-mediate TPM1 expression. Collectively, our work suggests that LINC01116 is a potent oncogene of CRC.

Recent researchers demonstrated that LINC01116 could induce proliferation and hinder apoptosis of osteosarcoma, epithelial ovarian cancer and breast cancer cells and that its overexpression is correlated with poor prognosis [15, 17, 25]. To date, the biological function and underlying mechanism of LINC01116 were little-known in CRC. Here, we analyzed the clinicopathologic features of each included patients and the detection revealed that LINC01116 was strongly expressed in CRC tissues, suggesting LINC01116 plays a pivotal role in CRC progression. Further, CCK-8 and colony formation assays uncovered that the capacity of CRC cell proliferation was observably enhanced in response to LINC01116 overexpression while inhibited by LINC01116 knockdown. Existing study unraveled that abundant expressions of LINC01116 potentiate glioma cell proliferation, invasion, migration, cycle and angiogenesis [19]. In our study, tube formation and scratch assays presented that LINC01116 can modulate angiogenesis in CRC. All these studies support our conclusion that LINC01116 was validated to play an oncogenic role in promoting cell proliferation, tumorigenesis and angiogenesis, and therefore can be considered as a biomarker for tumor diagnosis and clinical prognostic index for patients with CRC.

Tropomyosin (TPM), which contains TPM1, TPM2, TPM3 and TPM4 in mammals, is a major structural component of cytoskeletal microfilament [26, 27]. Recent research demonstrated that TPM1 as an anti-oncogene has been reported in many types of cancer [28–30]. Importantly, the involvement of TPM1 in regulation of CRC cell proliferation has been reported [31]. TCGA database revealed TPM1 expression was repressed in CRC tissues. Lowly expressed TPM1 in CRC cells were further evaluated by qRT-PCR and Western blot. Moreover, TPM1 was aberrantly expressed after LINC01116 overexpression and silencing, indicating LINC01116 facilitates CRC development through TPM1-dependent mechanisms. Silencing LINC01116 and TPM1 were achieved in CRC cells to measure cell proliferation and angiogenesis and the detection revealed that inhibition of LINC01116 and TPM1 in CRC cells can further enhance cell proliferation and angiogenesis than LINC01116 knockdown alone, suggesting that TPM1 may be regulated by LINC01116 in CRC cells. Collectively, these findings implicated the anti-oncogenes of TPM1 led us to explore the roles of LINC01116 upregulation in regulating TPM1 in CRC cells.

It has been shown that lncRNA guides EZH2 to its target sites by interacting with sequence-specific transcriptional factors [32, 33]. In our study, RNAInter predicted that LINC01116 can bind with EZH2, and EZH2 is a transcription factor of TPM1 which was analyzed by UCSC. Both tumor-suppressive and oncogenic effects of EZH2 have been reported in human malignancies [34]. TCGA database confirmed that EZH2 was overexpressed in CRC tissues and negatively associated with TPM1 expression. EZH2 is a specific H3K27me3 histone methyltransferase and EZH2 can promote the expression of H3K27me3 [35]. RNA pull-down supported that H3K27me3 was enriched in LINC01116-complex. Further, qRT-PCR and Western blot presented that EZH2 was overexpressed in CRC cells and RIP confirmed that LINC01116 could bind with EZH2. After LINC01116 was overexpressed, ChIP showed that EZH2-enriched TPM1 promoter increased significantly. We first proved that LINC01116 regulated EZH2 in CRC at a posttranscriptional level. To further dissect the functional role of EZH2 and TPM1 in the development of CRC, pcDNA3.1-EZH2, sh-EZH2 or sh-EZH2 + sh-TPM1 were transfected in CRC. The detection described that EZH2 negatively regulates TPM1 expression, and further results validated that EZH2 blocks TPM1 to promote CRC cell proliferation, and EZH2 knockdown on TPM1 to promote angiopoiesis were further confirmed by tube formation and scratch assays.

Additionally, we further confirmed our results in vivo experiments. qRT-PCR and Western blot revealed that sh-LINC01116 group had inhibited LINC01116 and EZH2 expressions, and increased TPM1 expression in tumor tissues of nude mice. H&E staining revealed that sh-LINC01116 group showed a massive tumor-cell necrosis in tumor tissues and had decreased tumor size. IHC presented that the levels of CD31 and Ki-67 in sh-LINC01116 group were reduced effectively than those in sh-NC group. Collectively, our in vivo assay demonstrated that silencing LINC01116 expression considerably restricted the tumorigenicity and angiogenesis of CRC cells in nude mice.

## Conclusion

In conclusion, our research uncovered a novel lncRNA LINC01116 that overexpressed in CRC was closely related to the survival and prognosis of CRC, which might provide an objective index for clinical differential diagnosis. Also, our findings revealed the pro-tumorigenesis role of LINC01116 in CRC, and the mechanism whereby LINC01116 promotes CRC proliferation and angiogenesis by enhancing EZH2 expression as a transcription factor to downregulate TPM1. Above all, LINC01116 has multiple biological functions in the occurrence of CRC, suggesting that LINC01116 may be a promising therapeutic approach for CRC.

## Data Availability

The datasets used or analyzed during the current study are available from the corresponding author on reasonable request.

## Abbreviations

CRC: Colorectal cancer
lncRNAs: Long non-coding RNAs
TPM1: Tropomyosin 1
HUVECs: Human umbilical vein endothelial cells
RIP: RNA immunoprecipitation
ChIP: Chromatin immunoprecipitation
SPF: Specific pathogen-free
DAB: Diaminobenzidine
ANOVA: One-way analysis of variance
